# Exploring Service Users’ Experiences of Activities Used During Their Occupational Therapy Treatment in South Africa

**DOI:** 10.3390/healthcare14162630

**Published:** 2026-08-20

**Authors:** Mahlako Makhubela, Eunice Seekoe, Jayne Donaldson, Frank Kronenberg, Lieketseng Ned, Elelwani Ramugondo

**Affiliations:** 1 Department of Occupational Therapy, Sefako Makgatho Health Sciences, Ga-Rankuwa 0208, South Africa; 2 Faculty of Health, Sport and Society, University of Stirling, Stirling FK9 4LA, UK; jayne.donaldson@stir.ac.uk; 3 Deans office, College of Human Sciences, University of South Africa, Pretoria 0002, South Africa; eseekoe@gmail.com; 4 Occupational Therapy, University of Cape Town, Cape Town 7700, South Africa; frank.kronenberg@gmail.com (F.K.); elelwani.ramugondo@uct.ac.za (E.R.); 5 Division of Disability and Rehabilitation Studies, Faculty of Medicine and Health Sciences, Stellenbosch University, Stellenbosch 7600, South Africa; lieketseng@sun.ac.za

**Keywords:** theories, models, frameworks, western paradigms, African knowledge systems, decolonisation, and cultural relevance

## Abstract

**Background/Objectives**: Occupational therapy theories, models, and frameworks appear to be developed in Western countries. The philosophical framework for occupational therapy in South Africa appears to have relied upon various models, predominantly stemming from Western and European origins, with vague and fleeting references to African knowledge systems and philosophies. The objectives of the study were to explore how service users understand occupational therapy following their engagement in occupational therapy services; service users’ perceptions of the activities used during occupational therapy; and service users’ participation in decisions regarding the selection of activities during occupational therapy. **Methods**: This study used an interpretive qualitative design. Purposive sampling was used to select participants. A total of 21 participants were interviewed before reaching data saturation; 30 min semi structured interviews were conducted. Data were collected by the research assistant. Data were analysed using inductive thematic analysis at the latent level. **Findings**: Four themes were derived from the data, which are misconceptions and vagueness, impact yet optimism, limited meaningful activities, and mostly therapist-directed activity choice. **Conclusions**: The findings highlight systemic barriers such as language of communication in occupational therapy practice and education. They also suggest ambiguity in the understanding of therapeutic goals by service users. In the findings of this study, it is also indicated that there seems to be a lack of meaningful activities selected for therapy. This is shown by the activities, such as the exercises, in which the participants expressed their participation during occupational therapy. The power dynamics, knowledge, and use of technology in occupational therapy practice were also highlighted in the findings.

## 1. Introduction

This paper is part of a larger doctoral study currently in progress at Sefako Makgatho Health Sciences University (SMU) and the University of Stirling (UoS). The title of the study was for a framework for decolonising activity selection training in Occupational Therapy curricula in the South African context. Occupational therapy is founded on the belief that human beings are occupational beings whose well-being depends on participation in meaningful activities [1]. This fundamental belief underpins the use of activities as a medium for both assessment and intervention in occupational therapy practice [2]. As such, occupational therapy training includes strategies for selecting activities, with an emphasis on ensuring that these activities are meaningful and person-centred [3]. Despite occupational therapy’s commitment to person-centred practice, approaches to activity selection have historically been shaped by Eurocentric epistemologies that privilege Western understandings of health, independence, productivity, and meaningful occupation. Consequently, activities selected within practice may not always reflect the cultural values, lived experiences, and occupational realities of service users from diverse contexts, particularly in the global South, including South Africa. Understanding of whether the activities selected for therapy are perceived by service users as meaningful, culturally relevant, and aligned with their own priorities remains limited. Although therapists are taught to select activities through clinical reasoning and alignment with service user goals, the perspectives of those receiving therapy (referred to as service users in this paper) on these activities remain under-represented in the literature. This disconnect raises important questions about the extent to which person-centred principles are realised in practice. Activities are understood as purposeful tasks or actions that are selected and structured to develop, restore, or enhance the functional performance of a person and participation in meaningful occupations [4,5].

As an occupational therapist and doctoral researcher, the primary author entered this research work with an appreciation of occupational therapy’s commitment to person-centred and meaningful practice. However, the author’s experience in clinical practice in South Africa prompted the author to critically question whether the activities routinely selected in occupational therapy were consistently experienced by service users as meaningful and culturally relevant. She observed instances in which service users expressed confusion, disengagement, or dissatisfaction with activities that had been selected to facilitate therapeutic goals, raising questions about whose understanding of meaningful occupation was informing clinical decision-making. This was evidenced in some cases where the end-product of the occupational therapy intervention was discarded immediately after the session, particularly paper-based activities such as greeting card making and origami. Although this behaviour may have reflected multiple factors, it suggests that some activities and their products were not perceived as personally valuable or relevant. This observation echoes concerns raised in the literature about the importance of meaningful occupations in promoting engagement and therapeutic outcome [6]. The existing literature has primarily explored the understanding of occupational therapy among other healthcare professionals and students [7,8,9], the role of occupational therapy within specific conditions and practice settings [10], and occupational therapists’ own perspectives on practice [11]. However, comparatively little attention has been given to the experiences of service users, causing empirical evidence examining service users’ own experiences of activity selection to remain limited, particularly within the South African context.

These observations did not constitute evidence for the findings of this study; rather, they motivated a systematic inquiry into the experience of service users themselves. Recognising that the author’s professional training was shaped largely by theories and practices of occupational therapy of predominantly western origin, the author approached this study through a reflexive and decolonial lens [12]. This required ongoing critical reflection on the author’s assumptions about activities, meaningful occupation, and clinical expertise, while intentionally privileging service user accounts as legitimate sources of knowledge [13].

Consequently, this study does not seek to evaluate the effectiveness of specific activities from the perspective of occupational therapists. Instead, it centres the experiences and meanings ascribed to activities by those receiving occupational therapy, with the aim of contributing to a more culturally responsive, collaborative, and contextually relevant occupational therapy practice [14].

Addressing this gap is important because occupational therapy increasingly advocates for collaborative, culturally responsive, and occupation-focused practice. Such approaches require not only that the therapists apply clinical reasoning, but also that service users actively participate in decisions about the activities used during therapy. By foregrounding service users’ experiences, this study contributes empirical evidence on how activities are understood, valued, and experienced by those receiving occupational therapy [15]. In doing so, it extends current discussions on person-centred practice by examining whether the activities selected in practice reflect the principles that underpin occupational therapy. The findings may inform the selection of therapeutic activities, strengthen collaborative clinical decision-making, and support the development of more culturally responsive interventions. Furthermore, the findings have implications for occupational therapy education by informing how activity analysis and activity selection are taught within professional training programmes.

The purpose of this study was to explore the experiences of occupational therapy service users regarding the activities used during their treatment sessions in South Africa. Specifically, the study sought to (1) explore how service users understand occupational therapy following their engagement in occupational therapy services; (2) explore service users’ perceptions of the activities used during occupational therapy; and (3) explore service users’ participation in decisions regarding the selection of activities during occupational therapy.

## 2. Materials and Methods

This study used an interpretive qualitative design to explore how users of the occupational therapy service understand and experience the activities used during the intervention. An interpretive qualitative approach seeks to understand the meanings individuals attribute to their experiences within their social and cultural contexts [16,17]. An interpretive qualitative design not only describes service users’ experiences but also interprets the meanings they attributed to the activities used during occupational therapy treatment within their social and healthcare contexts [18,19]. In this study, the design was informed by a decolonial lens, which recognised the experiences of service users as legitimate sources of knowledge to examine the cultural relevance and meaningfulness of activities within occupational therapy practice. This methodological orientation aligns with the use of reflexive thematic analysis at the latent level, which seeks to generate interpretive insights rather than merely provide descriptive summaries of participants’ accounts [20].

The study was conducted in the public health sector in Gauteng and North West provinces, South Africa, across four hospitals, with two hospitals selected from each province. These settings were deliberately chosen to ensure diversity in terms of language, ethnicity, and cultural background, providing a rich and varied sample.

A purposive sampling strategy was used to recruit participants who met the study’s inclusion criteria and were able to provide relevant information that aligned with the study aims. Eligible participants included users of occupational therapy services aged 20 years and older, with no identified cognitive impairments, irrespective of employment status. Both service users who were still receiving occupational therapy services and those who had been discharged were included.

The researcher identified recruitment sites and, in collaboration with occupational therapists in each participating hospital, identified potential participants from hospital records who met the inclusion criteria of the study. Occupational therapists acted solely as gatekeepers, facilitating access to eligible participants by screening records against inclusion criteria. They were not involved in obtaining informed consent, collecting data, or determining who ultimately participated in the study. Recognising the potential power relationship between occupational therapists treating and service users, several measures were implemented to minimise the risk of perceived duress or undue influence. After identifying eligible participants, the researcher called them and arranged a meeting with interested individuals independently from the treating therapist, explained the purpose and procedures of the study, answered any questions, and provided the participant information sheet.

Participation was entirely voluntary. Written informed consent was obtained by the researcher only after the participants had had sufficient time to consider participation and ask questions. Participants were reminded that they could decline participation or withdraw from the study at any stage without providing a reason and without adverse consequences for their ongoing care. Occupational therapists were not informed of the participants’ decisions regarding participation unless the participants chose to disclose this themselves, thus protecting confidentiality and reducing the potential for perceived pressure to participate.

Data were collected using semi-structured, face-to-face individual interviews, guided by an interview schedule (Supplementary Materials) developed by the researcher in accordance with the study’s research question and objectives. The interviews were held in a private room/office at each site without an interpreter since the research assistant is fluent in all four languages used to collect data: English, Sepedi, Setswana or isiZulu. Interviews were conducted by a research assistant who received prior training from the researcher to ensure familiarity with the study purpose, interview procedures, and interview guide.

The use of an independent interviewer was intended to minimise the potential influence of the researcher’s professional identity as an occupational therapist on participants’ responses. As the research assistant was not affiliated with the occupational therapy departments where the participants received treatment, the participants may have felt more comfortable expressing positive and negative experiences of occupational therapy without concern that their responses could influence their care or their relationship with their therapists.

This independence was considered particularly important given the study’s aim of eliciting authentic accounts of participants’ experiences of activities. Although the research assistant’s independence may have facilitated more open disclosure, it may also have influenced the data in other ways. As he was not an occupational therapist and had no prior involvement in the study, he may not have recognised opportunities to probe occupation-specific concepts or clinical terminology beyond the interview guide. To minimise this limitation, the researcher provided training on the purpose of the study, the interview schedule, interview techniques, and the use of appropriate follow-up probes to encourage participants to elaborate on their responses while avoiding leading questions. Regular debriefing sessions were held between the researcher and the research assistant throughout the data collection period to discuss the interview process, clarify emerging issues, and ensure consistency across interviews. Each interview lasted approximately 30 min.

With participants’ consent, interviews were audio-recorded and conducted in one of four official languages—English, Sepedi, Setswana, or isiZulu, depending on participant preference—to minimise language barriers. These language choices reflected the dominant languages spoken in the Gauteng and North West provinces. A total of 21 participants were interviewed. Data collection continued until it was observed that subsequent interviews consistently reiterated information that had already been identified in previous interviews. Rather than generating substantially new insights related to the experiences of the service users on activities selected for their therapy, later participants largely confirmed and expanded upon existing patterns of meaning. The recurrence of similar experiences and perspectives indicated that the data set was sufficiently rich and comprehensive to address the research objectives. Consequently, data collection was discontinued because additional interviews were unlikely to contribute meaningful new insights to the analysis.

The audio-recorded interviews were transcribed verbatim by the researcher through manual listening and transcription. Audio-recorded interviews conducted in Setswana, Sepedi, and isiZulu were transcribed verbatim and subsequently translated into English for analysis. The translation was undertaken with the intention of preserving the conceptual meaning of the responses of the participants rather than producing literal translations of words for words. Attention was paid to culturally specific expressions, idioms, and contextual meanings to ensure that the intended message was retained during the translation process.

Recognising that translation can result in the loss of linguistic nuance or culturally embedded meanings, several strategies were employed to minimise this risk. The researcher, who is the first Sepedi language speaker, reviewed each Sepedi translation transcript together with the original audio recordings to verify the accuracy and consistency of the translations. The researcher appointed a Setswana first speaker to verify the ones translated from Setswana and did the same for the ones translated to isiZulu. Where words, phrases, or concepts did not have direct English equivalents, translations prioritised conceptual equivalence over literal translation, and explanatory wording was incorporated where necessary to preserve the participants’ intended meanings. The transcripts were labelled using participant pseudonyms to protect anonymity, according to Creswell and Poth [17]. Data management involved creating multiple secure copies of recordings and transcripts, stored on different platforms to ensure accessibility and data safety.

Data were analysed using inductive thematic analysis at the latent/interpretive level. Analysis was conducted using NVivo version 14, following the step-by-step process outlined by Braun et al. [21] as follows:

- The researcher familiarised herself with the data by reading and re-reading it. Transcribing the records helped the familiarisation process.
- The researcher coded the data by identifying the important features and ideas from the data that are relevant to answering the research question and the objectives. This was done through Nvivo 14 to organise the codes and the quotes.
- The researcher examined the codes and collateral data to identify and derive themes inductively.
- The researcher reviewed the themes by checking them against a data set to verify that they answered the research question and helped to achieve the aim and the objectives of the study. The themes were also reviewed by combining and splitting them in order to finalise the themes and avoid repetition; then, subthemes were also derived at this stage.
- The researcher named the themes and described them. Names of the themes developed from the data set and the meanings the researcher attached to the data.
- The researcher then presented the data according to themes and subthemes.

Trustworthiness was established using the criteria proposed by Creswell and Poth [17], with strategies embedded throughout the analytical and interpretative process. Participant validation (member checking) was not undertaken in this study. Consistent with the interpretive qualitative design and Braun and Clarke’s approach to thematic analysis, the findings represent the researchers’ interpretive engagement with participants’ accounts rather than an objective account requiring participant verification. Participants’ perspectives may also change over time, and requesting validation of the researchers’ interpretations may not necessarily enhance the credibility of the analysis. However, credibility was enhanced through continuous engagement with the data and regular peer scrutiny. As the development of the coding and themes progressed, preliminary interpretations were presented during supervisory meetings, departmental research discussions, and review sessions of the School of Health Care Sciences. These discussions encouraged critical examination of coding decisions, challenged emerging interpretations, and refined theme development, thereby reducing the influence of researcher bias. Reflexive engagement throughout the analysis enabled the researcher to critically examine how her positionality as a Black South African woman, together with her professional training within the predominantly Western occupational therapy system that this study critically interrogates, may have shaped the interpretation of the data. Ongoing reflection supported awareness of these assumptions and helped ensure that the findings remained grounded in participants’ accounts while privileging their experiences as legitimate sources of knowledge.

Transferability was facilitated through the provision of rich and contextual descriptions of the study setting, participants, data collection procedures, and analytical processes. Detailed accounts of the research context, together with verbatim participant quotations presented within the findings, allow the reader to determine to what extent the findings may be applicable to similar clinical contexts.

The dependability was strengthened through a systematic and transparent analytical process. A code–recode procedure was undertaken by which the researcher independently coded the transcripts and repeated the coding process after a one-week interval. The two coding iterations were compared to assess consistency in code application and theme development, with any discrepancies prompting further engagement with the data before finalising the coding. Throughout the analysis, an audit trail was maintained using NVivo 14 software to document coding decisions, theme refinement, analytic memos, and methodological changes, thereby providing a transparent record of how interpretations evolved over time.

Confirmability was supported through ongoing reflexive practice and transparent documentation of methodological and analytical decisions. Reflexive notes and analytic memos were maintained throughout the study to record the rationale for coding choices, theme refinement, and interpretative decisions, enabling the researcher to distinguish participants’ accounts from personal assumptions. Peer debriefing with supervisors and academic colleagues provided opportunities to critically evaluate the coherence of the interpretations and the evidentiary basis of the themes. Collectively, these strategies improved the transparency, consistency, and trustworthiness of the analytical process by ensuring that the findings were grounded in the data and could be systematically traced throughout the study.

This study was part of a doctoral research project that received ethical approval from the Sefako Makgatho Health Sciences University Research Ethics Committee (SMUREC/H/388/2022:PG) and the University of Stirling Research Ethics Committee (Reference: 10477). Permission to conduct the study was obtained via the National Health Research Database, as well as the Gauteng and North West provincial health research databases.

Participation was voluntary and all participants were informed of their right to withdraw from the study at any time without providing a reason. Written informed consent was obtained prior to data collection. Confidentiality and anonymity were maintained by using pseudonyms and restricting access to the data to authorised individuals only. Confidentiality was also maintained by anonymising the sites/hospitals where the participants were obtained. Participants were assured that no identification information would be included in any reports or publications arising from the study.

The research data was securely stored on DataSTORRE (Stirling Online Repository for Research Data), with additional copies saved on an external drive and stored in a locked safe room within the Department of Occupational Therapy at Sefako Makgatho University.

## 3. Results

### 3.1. Demographic Data

Twenty-one participants were interviewed in the study. The ages of the participants at the time of data collection ranged from 20 to 66 years. Six participants were between 20 and 30 years old, six participants were between 30 and 40 years old, seven participants were between 40 and 50 years old, one participant was over the age 50 years, and one was over the age of 60 years. The study sample comprised 15 male participants and six female participants, resulting in a greater representation of male perspectives. Although purposive sampling was used to recruit participants who could provide rich accounts of their experiences, the gender distribution may have influenced the breadth of experiences captured. Consequently, the findings may be more reflective of the experiences of male occupational therapy service users within the study context and should be transferred cautiously to contexts where female service users predominate or where gender-related experiences of occupational therapy may differ. Eighteen participants identified as black and three participants identified as white. All participants were South African residents. Five participants were unemployed at the point of the data collection, five were self-employed (running small businesses such as street vendor, taxi owner, etc.), ten participants were employed in semi-skilled labour market, and only one participant was a pensioner.

All participants underwent occupational therapy rehabilitation for a period between 2 and 24 months, and most of the sessions were scheduled once a month. This was due to logistics and hospital overcrowding. All participants were attending occupational therapy as outpatients.

### 3.2. Themes Derived from the Data Set

Data were analysed using inductive reflexive thematic analysis at the latent level in NVivo version 14, following the analytical process described by Braun and Clarke [21]. Four themes and eight subthemes were generated, representing patterns of shared meaning across the experiences of the participants with occupational therapy services. The themes and subthemes are presented in Table 1 and discussed below with supporting participant quotations.

This phase of the study yielded four themes, presented below, as guided by Braun et al. [21].

The first theme derived from the data is misconceptions and vagueness. This theme focuses on the knowledge and understanding of occupational therapy as expressed by the participants after receiving occupational therapy services. It emphasises misconceptions, as well as superficial understanding of the profession. The subthemes are misconceptions despite partaking in occupational therapy and vague awareness of occupational therapy.

Some of the participants said they understood what occupational therapy is about after the therapist explained to them. However, most of these participants define occupational therapy with many misconceptions, confusing it with physiotherapy. Some of the participants expressed that they do not really know what occupational therapy is, or its purpose in health sciences, even though they have received occupational therapy treatment. Some participants continued throughout the interview by referring to occupational therapy as physiotherapy. The expressions of some of the participants are shown below:

*“From what I understand, occupational therapy is a process by which a person comes here… or when a person is injured and cannot use certain parts of their body… it’s a process that nurses and doctors use to help you by showing you exercises that you can use to heal.”*

Thomas.

*“It has to do with these trainings (exercises).”*

Jack.

*“OT works with treating your hands. They help you when you are injured by massaging you.”*

Lucas.

Some participants expressed that they understood what occupational therapy does and its role. However, most of the participants who expressed that they understood the role of occupational therapy seem to have very superficial and vague knowledge of this. The expressions of some of the participants are shown below:

*“My understanding of occupational therapy is exercises that are designed to help you regain functionality on whatever limb that was operated on. That is my understanding of occupational therapy.”*

Vusi.

*“Okay so, occupational therapy is not a physical… where you help someone physically. Obviously, that is the biggest part in helping someone physically with their body, or their hands. Or their arms or whatever they have to work with. But emotionally and mentally it plays as big a role as the physical, and that’s what they do.”*

June.

*“Occupational therapy is where they help us to get to work on our injured parts of the body so we can recover from accidents so that our limbs can work.”*

Tshidi.

The second theme derived is partial impact, yet optimism. This theme captures the experiences of participants that expressed the impact of occupational therapy on their lives. The subthemes derived under this theme are movement gains, yet not holistic and confidence boast and positivity.

Most of the participants reported that attending therapy sessions contributed to noticeable physical/movement progress. Some described improvements in limbs that were previously immobile, noting that they could now move to some extent. However, the expressions of the participants also suggested a subtle sense of dissatisfaction with the results. Phrases such as “they are trying” and “at least” implicitly conveyed that the gains achieved were limited or below their expectations. The experiences of some of the participants are shown in the expressions below:

*“So far, I think they are trying because the exercises I was given are improving my hand. It’s just here and there… I can even hold things whereas I couldn’t hold anything before.”*

Tshepo.

*“Yes. At first, I couldn’t walk at all. But at least now as I’m doing these therapy sessions I’m trying.”*

Thapelo.

*“If it weren’t for them helping me my hand wouldn’t be saved at all. They would have been amputated. So the role they played in this process to get this hand functional is very huge and they really helped. “*

June.

Some of the participants expressed that occupational therapy provided emotional and psychological benefits in addition to physical support. Therapy sessions were reported to boost confidence, restore positivity, and encourage mental engagement, reflecting that the intervention contributed not only to physical rehabilitation but also to improved emotional well-being and cognitive functioning. The experiences of some of the participants are shown in the expressions below:

*“They help us not only physically, but also empower our minds. Like you can do it. They give you back your confidence, you get me.”*

Abel.

*“This is positive, yes. It is bringing some positivity back. So, I am confident that this hand will work normally again after this operation.”*

Vusi.

*“So it actually makes people realise that what you do with your brain can do wonders in your whole life. That helps a person exercise their thinking. Engage your brain for better. For positive.”*

Matthew.

The third theme derived is limited meaningful activities. This theme focuses on the types of activities experienced by participants during the occupational therapy intervention. The subthemes derived from this theme are mostly exercises and children’s activities, as well as continuity at home.

Most of the participants said that the activities they participated in during occupational therapy treatment were mostly exercises. One of the participants stated that he participated in walking exercises. Most of the participants who had a history of sports expressed that the sessions reminded them of the exercises they used to do in those years. One participant said that she was performing many different tasks, such as picking up toys and coins from the table. Some expressed that the activities they performed were what they thought were children’s activities such as cutting and playing with marbles. Very few participants expressed that they participated in some meaningful activities once or twice. The experiences of some of the participants are shown in the expressions below:

*“They tell you to walk slowly until you get to the next point. Stretch your leg. Slowly bend and straighten it “Obakeng”*.

*“They gave me balls to squeeze so that my fingers do not get stiff all the time.. I bend them and stretch them… My legs as well. There are bars with which we use and balance so that my feet can move as before”.*

Noluthando.

*I thought what is this? You are seriously giving me marbles? I must play with marbles? You give me these small sticks telling me to remove them? It’s like children’s activities”.*

Lucas.

*“They make me hold this……... I get to hold lids and pegs. They make me lift things with some heavy weight with my injured hand.”*

James.

Most participants expressed that they do some sort of exercise for a certain number of times a day at home as instructed by an occupational therapist. One participant stated that he was expected to participate at home during laundry, especially the hanging of clothes. Some participants indicated that exercises introduced during hospital occupational therapy were expected to continue at home through prescribed home programmes. Although this approach promoted continuity of rehabilitation beyond the clinical setting, participants’ accounts suggest that home programmes largely replicated exercise-based interventions rather than incorporating meaningful occupations embedded within their daily home, family and community contexts. The experiences of some of the participants are shown in the expressions below:

*“I was supposed to do activities practically once a day for ten minutes at home until I return. Yes, once a day for ten minutes….. Like stretch it…... I stretch my hand to make sure that my fingers are straight. …….. Tomorrow I stretch again for ten minutes and then I am done for the day. Just like that. So it is like a part of exercise.”*

Abram.

*“Well, they had me put my arm in hot water. Obviously, they told me to do it at home as well….. And then I got a silicon ball that I was squeezing……. And then also rice... I had to put my hand in rice and try to pick up rice particles one by one to try and return the feeling in my fingers”.*

Susan.

*“Eh, mostly I was told that I can help my mother at home whenever she does laundry by hanging the clothes on the washing line.”*

The fourth theme that was derived is mainly therapist-directed activity choice. This theme focuses on the contribution that participants made in regard to the activities selected for their occupational therapy service as expressed by the participants. The subthemes derived from this theme are following instructions, some limited agency and following the expert’s lead.

Most of the participants described their role in therapy as primarily following the instructions of the occupational therapist, indicating limited involvement in decision-making about therapy activities. This was particularly evident among those who attended therapy for the first time, who reported not yet having the opportunity to contribute to their treatment planning. As a result, many participants assumed a passive role within the therapy process. Some participants also expressed challenges with certain activities, noting feelings of impatience or dissatisfaction when exercises were perceived as time-consuming or did not align with their personal preferences. These responses suggest a possible mismatch between the selected activities and the individual needs or characteristics. In contrast, a small number of participants demonstrated some level of engagement by providing feedback on the activities, such as reporting whether the exercises were helpful. Although these instances indicate emerging participation, the overall findings suggest that service user participation was limited, with most participants participating in therapy as recipients rather than as active contributors.

*“No. I have not had any contribution. I think it’s because this is my first time.”*

Jack.

*But some of the exercises they are making me do are actually not for me because I am more of a street person. I am impatient with those exercises because they take too long.”*

Vusi.

*“since I have been here, I have never had the opportunity to contribute”*

Tshidi.

*“Yes. You know, some of the activities were hard for me so I would refuse to do them but then they would show me; if you do this, if you do this, it’s going to be easy for you”.*

Thapelo.

*“I also gave them feedback on the exercises they gave me……..”.*

Thea.

*“No, I’ve only done the ones they gave me.”*

Obakeng.

*“I also gave them feedback on the exercises they gave me that I am doing them as told and it is helping me a lot”.*

Thea.

Most of the participants stated that they view occupational therapists as experts in what they do in therapeutic interventions. They expressed that this does not make them contribute to the selection of activities for treatment. Some of the experiences are expressed below.

*“I thought you guys were professionals because you gave me everything. I couldn’t even get enough time to think about anything else.”*

Lucas.

*“No. I did not have any contribution because, since I don’t know what I need to do to fully heal without damaging myself, all I could do was ask questions about what I can and cannot do to ensure that I don’t disturb the healing process.”*

Vusi.

## 4. Discussion

This study explored the experiences of occupational therapy service users in the activities used during their therapy sessions. Four interconnected themes emerged from the analysis, reflecting the participants’ understanding of occupational therapy, their perceptions of its impact, their experiences of the activities used during the intervention, and their participation in activity selection. Interpreted through a decolonial lens, these themes provide insight into how broader assumptions about rehabilitation and occupational therapy practice shaped participants’ experiences. The discussion considers the striking points emerging from the findings in relation to the existing literature and examines their implications for occupation-centred practice, service-user participation, and the delivery of culturally responsive occupational therapy services within the South African healthcare context.

### 4.1. Systemic Barriers in Occupational Therapy Practice and Education

The findings of this study indicate that many service users demonstrated a misunderstanding or only vague awareness of the occupational therapy profession, despite actively receiving services from qualified occupational therapists. This raises important questions about how the profession is communicated to service users and how knowledge about occupational therapy is constructed within clinical encounters. Ideally, engagement with rehabilitation services should provide service users with at least a basic understanding of the profession providing care. This phenomenon invites deeper reflection on the structural and contextual factors that can influence how occupational therapy is understood by service users.

One potentially important explanation for the limited understanding of occupational therapy by participants is the language used during clinical interactions. Although this issue was raised by only one participant and therefore did not constitute a distinct theme, it provides valuable insight into how language may influence service users’ understanding of the profession. As one participant reflected: “Because she wasn’t using my language to explain, I cannot really say what she is explaining.” This account suggests that language barriers can hinder the understanding of occupational therapy by service users, particularly when complex professional concepts are communicated in languages that are not their primary means of communication. Within the context of multilingual South African health care, this finding highlights the importance of culturally and linguistically responsive communication as part of occupation-centred practice.

In many South African occupational therapy training programmes, professional education is conducted primarily in English and, in some institutions, in Afrikaans. However, a large proportion of service users speak indigenous African languages as their first language. This linguistic gap can create significant challenges in communicating complex professional concepts, such as the scope and purpose of occupational therapy. As a result, therapists may struggle to translate professional terminology and theoretical concepts into forms that are accessible and meaningful to service users.

From a decolonial perspective, language cannot be understood merely as a neutral medium of communication. Wa Thiong’o [22] argues that language is deeply connected to culture, identity, and knowledge systems, shaping how individuals understand themselves and the world around them. In Decolonising the Mind: The Politics of Language in African Literature, Wa Thiong’o indicates that colonial languages imposed through education systems often function to privilege certain forms of knowledge while marginalising indigenous ways of knowing. When professional knowledge is transmitted primarily through colonial languages, it may create a disconnect between institutional knowledge and the lived experiences of local communities.

Another critical issue relates to the limited inclusion of service user perspectives in occupational therapy research. Much of the literature exploring perceptions of occupational therapy tends to focus on the views of healthcare professionals or students rather than the perspectives of service users themselves. Although such studies provide insight into the interprofessional understanding of occupational therapy, they risk overlooking the voices of the communities that the profession aims to serve. From a person-centred practice perspective, this absence is problematic, as occupational therapy philosophy emphasises collaboration, participation, and person-centredness.

However, if research primarily privileges professional viewpoints, the lived experiences and interpretations of service users remain under-represented. Whalley Hammell [14] argues that occupational therapy research has historically been dominated by professional and institutional perspectives, often neglecting the knowledge and experiences of those receiving services. Therefore, the present findings highlight the importance of centring the voices of service users in research to better understand how occupational therapy is perceived, experienced, and interpreted outside of the professional discourse.

The literature found focuses on the understanding of occupational therapy by other health sciences professionals, including undergraduate students [7,8,9]. Some studies focus on the role of occupational therapy with respect to certain conditions and fields of practice [10] and on the perspectives/perceptions of the occupational therapists themselves [23]. Olaoye et al. [7] concluded in their study that medical and health sciences undergraduates had poor to moderate knowledge about the occupational therapy profession. In his thesis report, Jackson [8] states that it is well established that occupational therapy is a widely misunderstood profession.

Beyond communication and research gaps, the findings also raise questions about how occupational therapists themselves understand and articulate their professional identity. Some therapists may also experience uncertainty or difficulty in explaining the profession in accessible terms. Occupational therapy has long been described as a profession that struggles to define and communicate its unique role within healthcare systems [24]. This challenge may be intensified in contexts where therapists must translate complex theoretical constructs such as occupation, participation, and meaningful activity into practical explanations that resonate with everyday experiences of service users. If therapists themselves find the profession difficult to define succinctly, these misconceptions may be transmitted to service users during clinical interactions.

These challenges may also be linked to broader historical and epistemological factors shaping the profession. Occupational therapy as a discipline emerged largely within Western contexts, and much of its foundational philosophy, theoretical models, and educational curricula were developed in Europe and North America. When such foundational philosophy and curricula are transferred to non-Western contexts without further research, they may not align with the cultural realities, values, and everyday activities of local communities. Scholars working from decolonial and Global South perspectives have argued that rehabilitation professions must critically examine how Western knowledge systems continue to shape practice in postcolonial societies [25].

Within this context, occupational therapists of colour may experience tensions between the professional knowledge acquired during training and the cultural practices and understandings of occupation embedded in their own communities. Educational programmes may require students to adopt theoretical frameworks and conceptualisations of occupation that originate from Western societies, which may sometimes conflict with local cultural understandings of meaningful activity and collective living. As Ramugondo and Kronenberg [25] noted, occupational therapy education in the Global South often reflects colonial legacies in which Western epistemologies dominate professional training. Consequently, therapists may find themselves having to unlearn their realities and try to align their lives with these realities.

This potential disconnect between the cultural background of the therapists and the dominant philosophical foundations of occupational therapy may influence how the therapists explain the profession to service users. If therapists themselves experience difficulty reconciling the frameworks of western occupational therapy with the realities of their communities, articulating the purpose and relevance of occupational therapy to service users could become challenging. In such situations, therapy may be experienced primarily as a series of exercises or clinical tasks rather than as an activity-based intervention aimed at enabling meaningful participation in daily life. This could partially explain why several participants in the present study associated occupational therapy mainly with physical exercises or rehabilitation activities similar to physiotherapy.

Together, these findings suggest that misunderstandings about occupational therapy may not be solely attributed to a lack of knowledge among service users. Instead, they reflect a complex interplay of linguistic barriers, limited service user-centred research, challenges in professional identity articulation, and the historical dominance of Western epistemologies within occupational therapy education and practice. Addressing these issues may require a more deliberate effort to develop culturally responsive communication strategies, incorporate service user perspectives into research, and critically engage with the decolonisation of occupational therapy curricula and practice in contexts such as South Africa.

### 4.2. Ambiguity in Service Users’ Understandings of Therapeutic Outcomes

The findings of this study reflect subtle expressions of dissatisfaction with therapy outcomes among some participants. Although improvements were acknowledged, phrases such as *“I think they are trying…” and “at least I can…”* suggest cautious recognition of progress while implying that results may not fully meet the expectations of participants. These expressions mirror a sense of partial satisfaction, where participants appreciate the efforts of the therapists but still perceive limitations in the outcomes of the intervention. From a decolonial perspective, such responses may also reflect power relations embedded within healthcare systems shaped by colonial histories. Healthcare environments often position professionals as experts and service users as passive recipients of care, which may discourage patients from openly critiquing services. As a result, dissatisfaction may be expressed indirectly through a cautious language that recognises professional authority while subtly conveying unmet expectations, a dynamic that aligns with the argument of Ramugondo and Kronenberg’s [25] that everyday healthcare interactions can reproduce broader social power relations.

The findings of this study indicate that participants mainly reported improvements related to movement rather than functional or occupational outcomes. Although participants described gains such as increased mobility of the limb or the ability to perform certain physical actions, these improvements were rarely linked to meaningful engagement in everyday activities or occupational performance. This is concerning given that the core mandate of occupational therapy is not merely to restore movement, but to enable individuals to participate in meaningful occupations that support health and well-being. The emphasis on movement improvements suggests that therapy may have been experienced primarily within a biomedical rehabilitation framework, where physical recovery is prioritised over participation in daily life roles. A study carried out in the Netherlands with the aim of investigating the outcomes of occupational therapy of servicer users affected by COVID-19 through several standardised and observational tests concluded that participants improved significantly in daily functioning Cup et al. [26], which seems to contrast the findings of the current study as it only reports movement improvement rather than functional outcomes.

According to the World Federation of Occupational Therapists [27], occupational therapy is a holistic and person-centred profession that aims to support health, well-being, and participation through engagement in meaningful activities. The profession recognises that occupational performance arises from the dynamic interaction between the person, the occupations they engage in, and the environments in which those occupations occur. This perspective highlights that rehabilitation should extend beyond physical recovery to address the broader contexts that shape an individual’s ability to participate in meaningful activities of everyday life.

### 4.3. Lack of Meaningful Activities Selected for Therapy

The findings of this study indicate that many of the activities used during therapy were based on exercise and resembled children’s activities such as cutting with scissors and picking up marbles. This raises questions about whether these activities are meaningful and relevant for adult service users within their specific social and cultural contexts. It also draws attention to those who have knowledge and assumptions that inform the design of therapeutic activities and whether these reflect the lived experiences and everyday realities of the people receiving services. When selecting activities for the provision of services, it is important for the therapists to recognise service users as active participants who can contribute valuable knowledge about their own needs, interests, and meaningful occupations. The findings of Hoshii et al. [28] showed improved psychiatric outcomes when service users selected their own activities. When individuals are involved in the selection of activities, it may reduce the hierarchical relationships between therapists and service users and promote a more collaborative therapeutic process. This approach may also improve motivation, engagement, and overall satisfaction with therapy.

The literature on the kind of activities selected for occupational therapy is limited locally and globally; the existing literature focuses on the effectiveness and creativity of activities used in occupational therapy rather than the types of activities used in therapy [28,29,30]. Connor et al. [30] emphasise the role of participation in meaningful activities in recovery, management of chronic conditions, and the promotion of health and wellbeing. Although tools such as the Activity Card Sort (ACS) are useful for measuring participation, they were developed in Western contexts and may not fully represent activities that are meaningful in other cultural settings. Therefore, it is important for therapists to consider adapting assessment tools and intervention strategies so that they better reflect local contexts, cultural practices, and the daily occupations of the communities they serve.

In general, this perspective suggests that occupational therapy should place greater emphasis on activities that are meaningful and relevant to the lives of service users and on involving them more actively in decisions about their therapy. Such an approach can support more inclusive, responsive, and person-centred therapeutic practices and address power issues in therapeutic relationships between service users and occupational therapists.

### 4.4. Power Dynamics, Knowledge and the Use of Technology in Occupational Therapy Practice

The findings of this study suggest that service users often do not perceive themselves as having equal power to influence the choice of activities used during their occupational therapy sessions. Many participants viewed the therapists as primary experts and believed that their role was primarily to follow the instructions provided by the therapist. This perception reflects the underlying power dynamics within therapeutic relationships, where therapists are positioned as knowledge holders, while the knowledge of service users of their own lives, experiences and daily occupations is undervalued. When service users are not actively encouraged to participate in decision making regarding their therapy, the therapeutic process may unintentionally reproduce hierarchical relationships that limit collaboration and shared decision making.

Occupational therapy has long emphasised person-centred practice, which aims to recognise service users as active partners in therapy rather than passive recipients of care. Tondora et al. [31] argue that person-centred practice requires therapists to consider the unique experiences, contexts, and priorities of service users when planning interventions. Similarly, Restal and Egan [32] describe person-centredness as a process that views the individual as being embedded within broader social and environmental systems and emphasises collaboration during assessment, intervention, and evaluation. This suggests that service users should be meaningfully involved in decisions regarding activities used in therapy and goals rather than merely receiving interventions.

However, research indicates that service users do not always experience occupational therapy as genuinely collaborative. For example, a priority-setting initiative conducted by the James Lind Alliance reported that people who access occupational therapy services sometimes feel that their perspectives are not fully incorporated into therapy planning. These findings highlight an important gap between the theoretical commitment to person-centred practice and the realities experienced by some service users [33].

Another noteworthy observation emerging from this study is related to the growing role of digital platforms in shaping how service users engage with their rehabilitation. Although raised by a single participant and, therefore, not constituting a distinct theme, this account provides valuable insight into how service users may seek alternative sources of information when their rehabilitation needs or expectations are not fully met. As one participant explained: *“That is why I am saying it can be frustrating*. *I end up checking on YouTube and end up doing other things that were prescribed by the hospital.”* This observation illustrates that some service users may exercise agency beyond the clinical encounter by independently seeking rehabilitation information and strategies [34]. Digital platforms like YouTube have become increasingly accessible sources of health-related information, including rehabilitation exercises and educational resources.

Previous research has shown that many patients search the internet for information about their health conditions before or after clinical consultations [34]. Although access to digital information can improve self-management and enable service users to participate more actively in their recovery, it also raises important questions about the quality, safety, and appropriateness of information obtained from unregulated online sources.

A recent analysis of stroke rehabilitation videos found that while some videos provide useful educational information, others are low quality or incomplete [35]. Studies examining rehabilitation information on YouTube for other conditions have similarly reported that a large proportion of videos contain unreliable or poor-quality information, highlighting the risks associated with unsupervised online learning [34]. At the same time, researchers acknowledge that when high-quality videos are developed by healthcare professionals, digital platforms can become useful educational tools that support service user learning and home rehabilitation [36].

The growing use of online rehabilitation platforms raises important questions for occupational therapy practice. On the one hand, access to online resources can empower service users by enabling them to seek information and participate more actively in their recovery. However, it may also reflect gaps in communication between therapists and service users or a lack of opportunities for service users to participate meaningfully in treatment planning. If the activities used in therapy were more closely aligned with the daily occupations and personal goals of the service user, the service user might feel less of a need to seek external sources for guidance.

This study has several limitations that should be considered when interpreting the findings. The study was conducted in selected public hospitals in the Gauteng and North West provinces, and the findings therefore reflect the experiences of occupational therapy service users within these specific public healthcare contexts. As an interpretive qualitative study employing purposive sampling, the aim was to gain an in-depth understanding of participants’ experiences rather than produce findings that are statistically generalisable.

Participants’ accounts were based on their personal experiences and recollections of occupational therapy sessions, which may have been influenced by individual interpretation and memory. However, conducting interviews in participants’ preferred languages facilitated rich descriptions and supported the authentic expression of their experiences.

Finally, while the findings were interpreted through a decolonial lens, the interpretations are grounded in participants’ narratives and should be understood within the context in which the study was undertaken. Future research involving a broader range of healthcare settings, provinces, and stakeholder groups could extend the understanding of activity selection and contribute to the development of more contextually responsive occupational therapy practice.

## 5. Conclusions

This study provides insight into how occupational therapy service users experienced the activities used during occupational therapy sessions within selected public hospitals in South Africa. Participants’ accounts suggest that their understanding of occupational therapy was shaped by communication practice and the nature of the activities provided as well as the limited involvement in selecting the activities or service provision. Many participants associated therapy primarily with physical rehabilitation and reported limited involvement in selecting therapy activities, highlighting opportunities to strengthen collaborative, occupation-centred practice. Interpreted through a decolonial lens, these findings underscore the importance of contextually responsive communication and approaches that recognise and value service users’ lived experiences and knowledge.

The findings have important implications for occupational therapy practice, education, and research. Occupational therapists should adopt more collaborative and contextually responsive approaches to activity selection that promote meaningful participation in everyday life. Occupational therapy education should strengthen students’ cultural and linguistic responsiveness while encouraging critical reflection on dominant knowledge systems. Future research should explore service users’ experiences across diverse contexts to further inform culturally responsive, occupation-centred practice.

The findings should be interpreted in light of the study’s context, as participants were recruited from selected public hospitals and their experiences may not reflect those of service users in other healthcare settings. Further research across diverse contexts is warranted to deepen the understanding of activity selection and support the development of culturally responsive occupational therapy practice.

## Figures and Tables

**Table 1:** Themes and subthemes.

Themes	Subthemes
**Misconceptions and Vagueness**	Misconceptions Despite Partaking in Occupational Therapy
	Vague Awareness of Occupational Therapy
**Partial Impact, Yet Optimism**	Movement Gains, Yet Not Holistic
	Confidence Boast and Positivity
**Limited Meaningful Activities**	Mostly Exercises
	Children’s Activities
	Continuity at Home
**Mostly Therapist-Directed Activity Selection**	Some Limited Agency
	Following Experts’ Lead

## Data Availability

Research data were securely stored on DataSTORRE (Stirling Online Repository for Research Data), with additional copies saved on an external drive and stored in a locked safe room within the department at SMU and is available upon request.

## Supplementary Materials

The following supporting information can be downloaded at: https://www.mdpi.com/article/10.3390/healthcare14162630/s1.

## Abbreviations

The following abbreviations are used in this manuscript:

DataSTORRE	Stirling Online Repository for Research Data
SMU	Sefako Makgatho Health Sciences University
WFOT	World Federation of Occupational Therapy
