# Supplementary material for: Exploring Service Users’ Experiences of Activities Used During Their Occupational Therapy Treatment in South Africa
Source: Healthcare (Basel). 2026 Aug 20;14(16):2630. doi: 10.3390/healthcare14162630 (PMC13511970; doi:10.3390/healthcare14162630)
Supplement: Supplementary file 1 [file healthcare-14-02630-s001.zip › healthcare-4391549-supplementary.pdf]

DATA COLLECTION TOOL: Phase 3: Interview Schedule

Background Information

Participant pseudoname: ……………………………………………

Date: ………………………… Age: ……………………..

Gender: ……………………… Race: ………………………

Diagnosis: ………………….Duration of rehabilitation: …………………………………

1. **What is your understanding of occupational therapy based on your experiences?**

Probe: What do you think the purpose of occupational therapy was?

2. **Can you describe the activities that you participated in during your occupational therapy treatment sessions?**

Probes:

- What kinds of activities did you do?
- How did you experience these activities?
- Which activities stood out to you, and why?

3. **How did you feel about the activities that were used during your occupational therapy treatment?**

Probes:

- Did you enjoy them?
- Did they seem relevant to your life?
- Were any activities meaningful or unhelpful?

4. **In what ways, if any, did these activities help you during your recovery or return to your everyday life?**

Probe: Can you give an example?

5. **Were you involved in deciding which activities would be used during your occupational therapy treatment?**

Probes:

- How were the activities selected?
- Did your therapist ask for your preferences?
- Would you have liked greater involvement?

6. **Is there anything else you would like to share about your experiences of the activities used during your occupational therapy treatment?**
